# Induction of Susceptibility to Disseminated Infection with IgA1 Protease-Producing Encapsulated Pathogens Streptococcus pneumoniae, Haemophilus influenzae Type b, and Neisseria meningitidis

**DOI:** 10.1128/mbio.00550-22

**Published:** 2022-04-14

**Authors:** Mogens Kilian, Steffen Husby, Jesper Andersen, Zina Moldoveanu, Uffe B. Skov Sørensen, Jesper Reinholdt, Hervé Tettelin

**Affiliations:** a Department of Biomedicine, Aarhus University, Aarhus, Denmark; b Department of Pediatrics, Aarhus University Hospital, Aarhus, Denmark; c Hans Christian Andersen Children’s Hospital, Odense University Hospital, Odense, Denmark; d Department of Pediatrics, Nordsjællands Hospital, Københavns Universitetshospital, Hillerød, Hillerød, Denmark; e Department of Microbiology, School of Medicine, University of Alabama at Birmingham, Birmingham, Alabama, USA; f Department of Microbiology and Immunology, Institute for Genome Sciences, University of Maryland School of Medicine, Baltimore, Maryland, USA; Emory University School of Medicine

**Keywords:** *Streptococcus pneumoniae*, *Neisseria meningitidis*, *Haemophilus influenzae*, meningitis, susceptibility, IgA1 protease, capsule, antibodies, *Escherichia coli*

## Abstract

Streptococcus pneumoniae, Neisseria meningitidis, and Haemophilus influenzae are the principal causes of bacterial meningitis. It is unexplained why only occasional individuals develop invasive infection, while the vast majority remain healthy and develop immunity when encountering these pathogens. A capsular polysaccharide and an IgA1 protease are common to these pathogens. We tested the hypothesis that patients are primed to susceptibility to invasive infection by other bacteria that express the same capsular polysaccharide but no IgA1 protease. Thereby, the subsequently colonizing pathogen may protect its surface with IgA1 protease-generated Fab fragments of IgA1 devoid of Fc-mediated effector functions. Military recruits who remained healthy when acquiring meningococci showed a significant response of inhibitory antibodies against the IgA1 protease of the colonizing clone concurrent with serum antibodies against its capsular polysaccharide. At hospitalization, 70.8% of meningitis patients carried fecal bacteria cross-reactive with the capsule of the actual pathogen, in contrast to 6% of controls (*P* < 0.0001). These were Escherichia coli K100, K1, and K92 in patients with infection caused by H. influenzae type b and N. meningitidis groups B and C, respectively. This concurred with a significant IgA1 response to the capsule but not to the IgA1 protease of the pathogen. The demonstrated multitude of relationships between capsular types and distinct IgA1 proteases in pneumococci suggests an alternative route of immunological priming associated with recombining bacteria. The findings support the model and offer an explanation for the rare occurrence of invasive diseases in spite of the comprehensive occurrence of the pathogens.

## INTRODUCTION

Streptococcus pneumoniae, Neisseria meningitidis, and Haemophilus influenzae constitute the principal causes of bacterial meningitis in humans. The capsular polysaccharide of these pathogens is a key virulence determinant and forms the basis of successful vaccines. Genetic mechanisms for up- and downregulated expression of the capsular polysaccharides evolved in all three pathogens, and the polysaccharides occur in multiple structurally distinct versions. In S. pneumoniae, more than 100 distinct capsular polysaccharide structures have been identified (1). Exchange of parts of the *cps* gene locus responsible for the capsular biosynthesis with other members of the species as well as with related commensal Streptococcus species is a frequent occurrence leading to change of capsular serotype (2, 3). N. meningitidis may express the capsular polysaccharide as one of 13 distinct structures (A, B, C, D, E, H, I, K, L, W, X, Y, and Z) termed serogroups, of which six (A, B, C, W, X, and Y) may cause invasive infections (i.e., meningitis and/or septicemia) (4). Among these, a distinct evolutionary lineage that expresses the serogroup A capsule is causing epidemics primarily in sub-Saharan Africa and in China (5). In Europe and North America, serogroups B and C account for the majority of cases, while cases of serogroup A have disappeared since 1970 (6, 7). Meningitis due to H. influenzae is almost exclusively due to clones expressing a serotype b capsule, one out of the six structurally distinct capsular polysaccharides (serotypes a through f) that may be expressed by particular evolutionary lineages of this species (8).

In addition to the regulated expression of a capsular polysaccharide, an immunoglobulin A1 (IgA1) protease is common to all three principal causative agents of bacterial meningitis (9, 10). Human IgA includes the two subclasses IgA1 and IgA2. IgA1 vastly predominates both in systemic and in respiratory mucosal compartments (11). The IgA1 proteases are endopeptidases that cleave a post-proline peptide bond (Pro-Ser or Pro-Thr) within the heavily glycosylated and elongated hinge region of human IgA1. Neither human IgA2 nor IgA from any animal species, apart from IgA1 from humanoid primates, is cleaved (12). Being serine proteases in H. influenzae and N. meningitidis and metalloproteases in S. pneumoniae, the IgA1 proteases constitute a striking example of convergent evolution of the ability to inactivate functions of the principal immunoglobulin isotype in the upper respiratory tract (12). The monomeric Fab fragments, released by IgA1 protease activity, retain their antigen-binding capacity (13). However, elimination of the Fc part negates antibody-mediated agglutination, phagocytosis, and trapping of secretory IgA1 (S-IgA1)-coated bacteria to the mucus layer lining the epithelial surface of the respiratory tract (14–18).

Apart from causing invasive infection, the three pathogens also occur as temporary colonizers of the upper respiratory tract of healthy humans. The pneumococcal carrier state in children is very frequent, often exceeding 50% at a given time (19, 20). In adults, carriage is significantly less frequent but increases among those living with children (21). Meningococci differ from that pattern as carriage is unusual in infants and children (22), whereas the carriage rate in young adults and individuals in semiclosed communities, including schools, universities, and military camps, may be 25 to 70% (23, 24). In the African meningitis belt, carriage prevalence increases from low prevalence in infants (approximately 0.7% in the rainy season) to a broad peak of 1.94% at age 10, then decreases in adolescence (25). Local vaccination programs directed at selected capsular polysaccharides affect carriage rates of individual serotypes/serogroups of both S. pneumoniae and N. meningitidis (26, 27). This is the case also for H. influenzae serotype b (Hib), which has become rare in countries with Hib vaccination. According to a recent review, the average carriage of H. influenzae type b among children in mainland China, which does not use systematic Hib vaccination as part of the national immunization program, is 5.9% (28). This is similar to rates in Western countries before introduction of Hib vaccination, although carrier rates in selected populations, including day care centers, may be higher. Carriage of Hib in adults is rare (29).

Apart from differences related to distinct pathogenic potential of serotypes and the genetic backbone of clones (8, 30–32), it remains unexplained why some individuals develop invasive infection, while the vast majority that become colonized remain healthy and develop immunity when encountering one of the three pathogens.

Protection against invasive infections by the three pathogens achieved by vaccination or by asymptomatic carriage is closely associated with serum IgG antibodies to the capsular polysaccharide. In addition, both asymptomatic carriage and infections with meningococci induce high levels of serum IgG antibodies to the IgA1 protease that do not decline over a 5-year period, in contrast to antibodies to the capsular polysaccharide, which rapidly decline (33, 34). However, despite overall genetic similarity of IgA1 protease genes among strains of N. meningitidis and H. influenzae (12), both genetic polymorphism and epitope heterogeneity exist within the individual species (35). Among N. meningitidis strains, two IgA1 protease cleavage types exist: type 1 cleaves a prolyl-seryl bond, and type 2 cleaves a prolyl-threonyl bond. Type 1 is almost exclusively associated with epidemic strains. In addition, assays using enzyme-neutralizing antibodies raised in rabbits identified five different IgA1 protease inhibition types among 133 meningococcal isolates (36). In H. influenzae serotype b strains, similar analyses show remarkable conservation of the IgA1 protease despite significant diversity among other members of the species (35). Results of analyses of IgA1 proteases of S. pneumoniae reflect the extensive genetic diversity of that species. Among 102 isolates representing the capsular serotypes most frequently associated with invasive infections, 17 different inhibition types were distinguished using antisera raised in rabbits (37). Previous studies of the antigenicity of IgA1 proteases in humans detected antibodies to a single purified meningococcal IgA1 protease applied as the antigen in an enzyme-linked immunosorbent assay (ELISA) (33, 34). However, the diversity of epitopes to which neutralizing antibodies may react, combined with the presence in the molecule of sequence repeat structures that likely function as an immunological decoy mechanism, means that levels of antibodies to a particular purified IgA1 protease do not necessarily reflect the potential for enzyme neutralization *in vivo*.

It has been an enigma how the three pathogens benefit from cleaving IgA1, as enzyme-neutralizing antibodies to the IgA1 protease conceivably emerge synchronously with the induction of IgA1 antibodies to surface epitopes of the bacteria, primarily capsular polysaccharides. Considering this, we presented a hypothetical model for the function of IgA1 proteases in the pathogenesis of invasive infections due to S. pneumoniae, N. meningitidis, and H. influenzae (38). According to this hypothesis, the key is induction of anti-capsular IgA1 antibodies prior to the induction of inhibitory antibodies to the IgA1 protease as a consequence of time-shifted acquisition of two different bacteria expressing the same capsular polysaccharide. Induction of preexisting IgA1 antibodies by the initially colonizing bacteria that do not express an IgA1 protease enables the subsequently colonizing pathogen to cleave the IgA1 antibodies and to take advantage of the released Fab fragments directed against its capsule. Here, we present findings from a series of experiments designed to evaluate this hypothesis. The model may provide an explanation of why most carriers remain healthy, while only occasional immunologically primed individuals develop invasive infection.

## RESULTS

### Colonization with N. meningitidis induces enzyme-neutralizing antibodies to IgA1 proteases.

Previous studies demonstrated induction of serum antibodies reacting with a purified serogroup A meningococcal IgA1 protease during asymptomatic carriage and infection (33, 34). To what extent these antibodies are enzyme neutralizing is not known. Using N. meningitidis as the model, we analyzed the induction of serum antibodies with the ability to inhibit the IgA1 protease of the individual autologous meningococcal isolates from military recruits who became colonized during the initial 2 weeks of enlisting. The levels of neutralizing antibodies were compared to those in persistent carriers or persistent noncarriers. In the latter group, we measured enzyme-neutralizing antibodies to the IgA1 protease of N. meningitidis strain HF13, which induces inhibitory antibodies against the entire spectrum of meningococcal and gonococcal IgA1 proteases when injected into rabbits (36).

**FIG 1:**
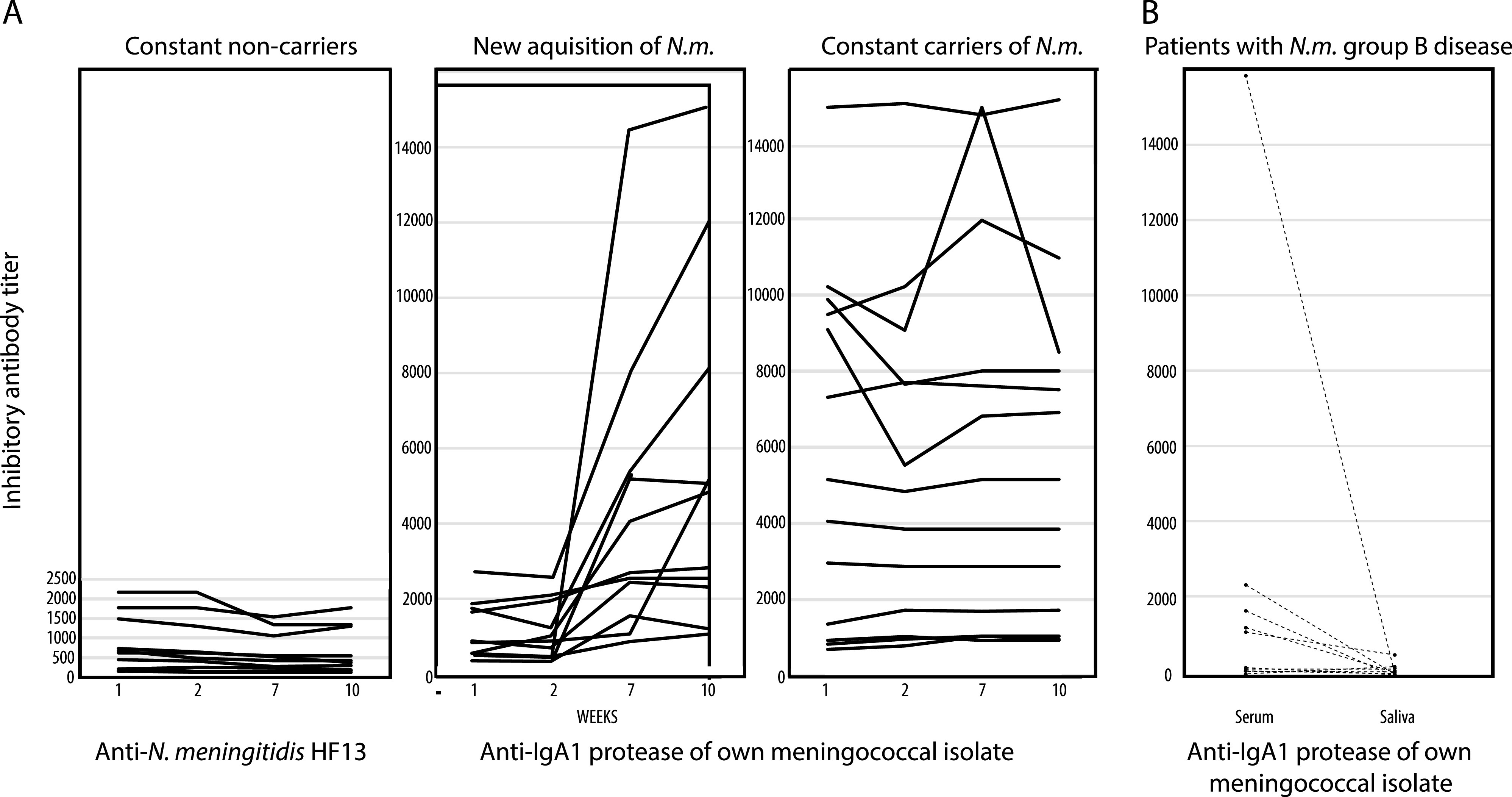
Inhibitory antibodies to activity-calibrated IgA1 proteases of individual N. meningitidis isolates. (A) Inhibitory serum antibodies to IgA1 proteases of N. meningitidis (*N.m.*) colonizing military recruits during the first 10 weeks of enrollment. Data are shown for constant noncarriers, recruits who became colonized during the first 2 weeks, and constant carriers (see Materials and Methods). (B) Neutralizing antibodies in serum and saliva samples collected upon hospitalization of 10 patients with N. meningitidis group B meningitis.

We observed a significant increase in inhibitory antibody titers in all of the 11 subjects who became colonized during the initial 2 weeks of observation (Fig. 1). A mean of an 8-fold increase between the initial and last samples (range, 1.4 to 28.3) was observed (increase from initial to last sample, *P = *0.0033). In contrast, inhibitory antibody titers were constant in the group of 14 who consistently carried the same clone (mean ratio between first and last measurements, 1.0; range, 0.3 to 1.6; *P = *0.92) whereas a limited decline was observed among the 11 persistent noncarriers (mean ratio between first and last measurements, 0.8; range 0.5 to 1.2; *P = *0.014). The titers observed in the last sample in persistent carriers (mean titer, 1,926) were significantly higher (*P = *0.0002) than those in the persistent noncarriers (mean titer, 607).

The results show that, concurrent with induction of serum antibodies against the capsular polysaccharide (34, 39–41), acquisition of meningococci in the pharynx induced a significant response of inhibitory antibodies against the IgA1 protease of the colonizing clone. Interestingly, even high levels of inhibitory antibodies apparently did not prevent or terminate colonization.

### Cross-reactive intestinal bacteria.

We then tested the hypothesis that patients developing invasive infections with H. influenzae type b or N. meningitidis encounter prior intestinal colonization with bacteria with capsules that cross-react with that of the pathogen. A total of 131 patients with invasive infections with one of the two pathogens and 233 controls with a matching age range, including patients with infections caused by other identified bacteria, were examined.

During the 3-year sampling period, we obtained fecal samples, bacterial blood isolates, and whenever possible, samples of blood and saliva from 49 patients in Denmark with invasive meningococcal disease (meningitis and/or septicemia): 41 with serogroup B and 8 with serogroup C meningococci (Table 1). The sampling period coincided with the introduction of the Hib vaccine as part of the Danish children’s vaccination program (June 1993). Therefore, samples from only eight patients with H. influenzae type b meningitis were obtained before virtual eradication of the disease in Denmark. To compensate, we analyzed fecal samples from 74 Finnish patients with invasive H. influenzae serotype b infection collected during 1985 and 1986 (42).

**TABLE 1:** Clinical diagnoses, etiologies, ages, and origins of 131 patients included in the study

Clinical diagnosis	Pathogen	Age		Origin	No. of patients (*n* = 131)
		Range	Median		
Meningitis	H. influenzae serotype b	2 to 72 mo	14 mo	Finland	52
Epiglottis		16 to 94 mo	51 mo	Finland	15
Septicemia		7 to 21 mo	14 mo	Finland	2
Cellulitis		8 to 19 mo	11 mo	Finland	5
Meningitis		4 mo to 17 yr	16 mo	Denmark	8

Meningitis/septicemia	N. meningitidis serogroup B	7 mo to 56 yr	9 yr, 9 mo	Denmark	41
	N. meningitidis serogroup C	8 mo to 20 yr	14 yr	Denmark	8

**FIG 2:**
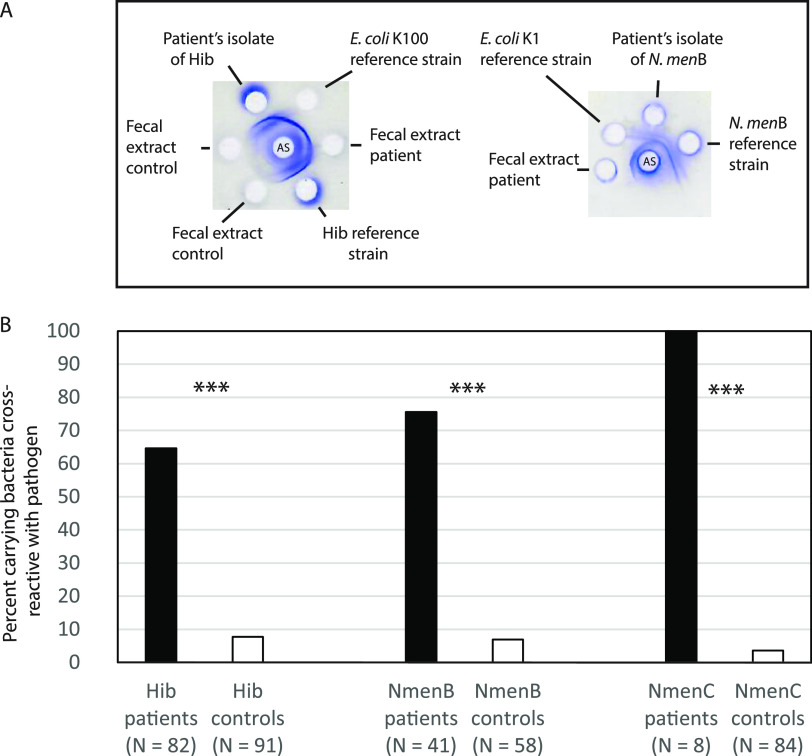
(A) Demonstrations by double-immunodiffusion assay of antigenic identity of fecal extract and isolated E. coli K100 reference strain to blood isolate of H. influenzae serotype b (Hib) (left) fecal extract and isolated E. coli K1 reference strain to blood isolate of N. meningitidis serogroup B (*N.men*B). The center wells contain antiserum (AS) against H. influenzae serotype b and N. meningitidis serogroup B, respectively. (B) Frequency of detection by double immunodiffusion of fecal bacteria cross-reactive with invasive H. influenzae serotype b and N. meningitidis serogroups B and C in patients with meningitis and septicemia and age-matched controls. ***, *P* < 0.001.

Immunodiffusion analysis of heated fecal sample extracts with the respective antisera against the capsular polysaccharides of H. influenzae type b and N. meningitidis (Fig. 2A) revealed the presence of antigens cross-reactive with the capsule of the actual pathogen in 70.8% of the patients, in contrast to 6% of the controls (Fig. 2B). The difference between patients and controls is highly significant (*P* < 0.0001).

From antigen-positive fecal samples, bacteria cross-reactive with the pathogen were successfully isolated as follows. (i) Among seven antigen-positive samples from Danish Hib infected patients, five yielded growth of Escherichia coli K100. (Due to extensive storage at −20°C, we did not attempt to isolate bacteria from Finnish samples.) (ii) E. coli K1 was isolated from 21 of 31 cases of N. meningitidis group B disease. (iii) E. coli K92 was isolated from four of 8 cases of N. meningitidis group C disease.

To exclude that the detected fecal cross-reaction was due to intestinal colonization with the actual pathogen, we attempted to recover H. influenzae type b and N. meningitidis groups B and C on selective media. None of the fecal samples yielded growth of the pathogen.

### Detection of antibodies against capsular polysaccharides and IgA1 protease.

Having demonstrated the presence of gut bacteria with cross-reactive capsules in the majority of meningitis patients, we then determined the levels of serum and salivary antibodies against the capsule and the IgA1 protease of the autologous blood isolate. To be able to relate the data to levels of neutralizing antibodies to IgA1 proteases detected in military recruits, we selected samples from patients with meningitis caused by serogroup B meningococci.

**FIG 3:**
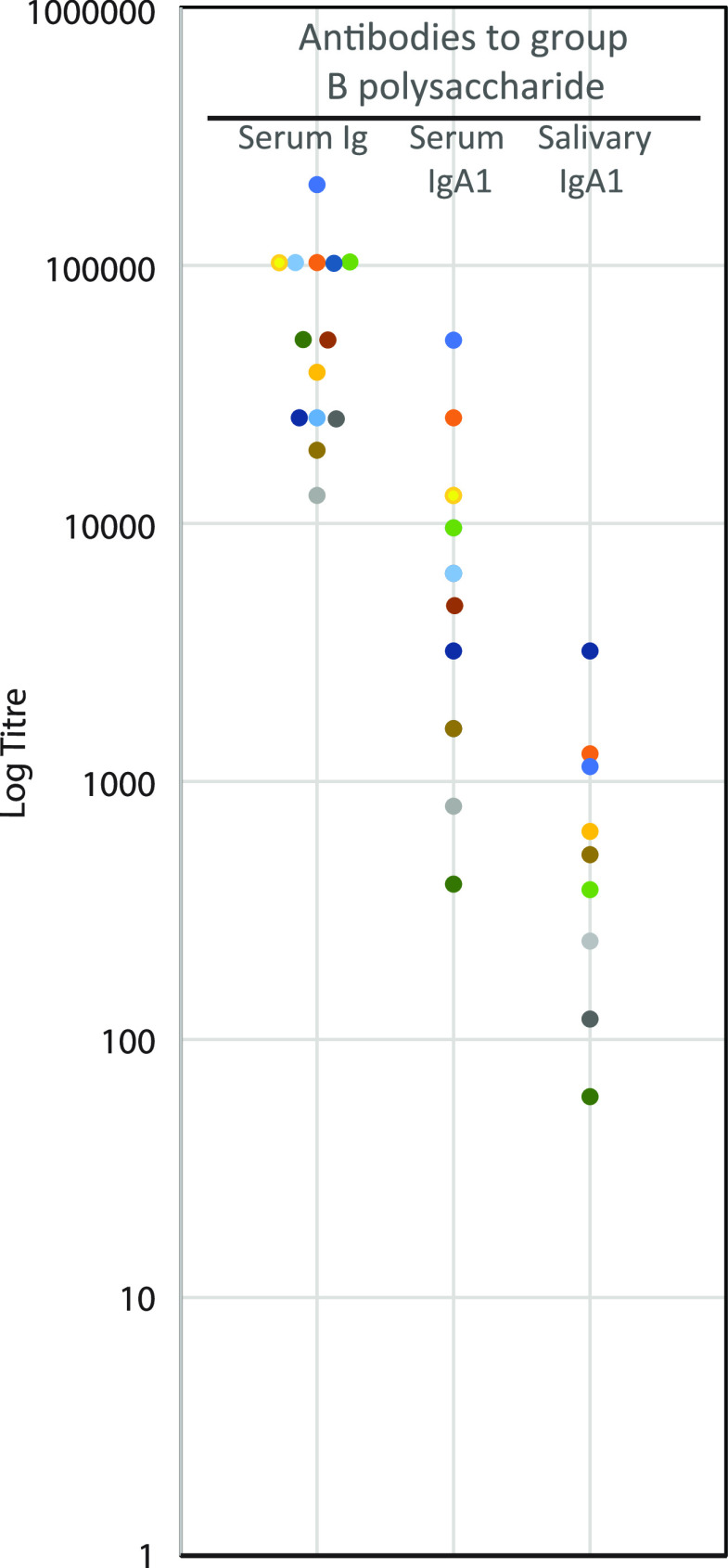
Endpoint titers of antibodies in serum (total immunoglobulins [Ig] and IgA1) and in saliva (S-IgA1) against capsular polysaccharide of N. meningitidis group B in 16 patients with invasive infection caused by this pathogen. The individual colors represent each of the 16 patients.

Samples of serum and saliva were collected from the meningitis patients whenever possible and as soon after admission to the hospital as possible. The samples were collected on the day of hospitalization or up to 5 days later (range, 0 to 5 days; median, 1 day). Figure 3 shows the levels of antibodies in serum and saliva against the serogroup B capsular polysaccharide of N. meningitidis. The figure demonstrates significant levels of anti-serogroup B antibodies in serum immunoglobulins (Ig) and IgA1, as well as in salivary IgA1, although at a lower level.

In 10 of the patients, we also quantitated the levels of antibodies in serum and saliva that neutralized the IgA1 protease of the infecting meningococcal strain. The assay was the same as that employed to quantitate neutralizing antibodies against individual activity-calibrated IgA1 proteases of autologous isolates from military recruits. Figure 1 shows the comparison. The levels of neutralizing antibodies in serum (Fig. 1B) were comparable to those observed in military recruits who were constant noncarriers (Fig. 1A). One exception (titer of 15,884) was a serum sample from a patient from whom the sample was collected 5 days after hospitalization. Surprisingly, the high titer of antibodies in serum of this patient was not reflected in the level of mucosal antibodies, as demonstrated in saliva.

### Genetic and antigenic diversity of IgA1 proteases among S. pneumoniae capsular serotypes.

Partly for logistical reasons (i.e., because of the large number of pneumococcal capsular polysaccharide structures [>100]), we did not attempt to identify cross-reactive bacteria in fecal samples of patients with pneumococcal meningitis. The genome of S. pneumoniae is extremely plastic due to its natural transformation competency and possession of numerous mobile genetic elements that facilitate genetic exchange by both transformation and conjugation. Therefore, we hypothesized that preexistence of IgA1 antibodies to the capsular polysaccharide of a particular serotype of S. pneumoniae clone that causes invasive infection may not necessarily be induced by another bacterial species. Rather, such antibodies may be induced by prior colonization with another clone of the same serotype of S. pneumoniae that expresses a distinct IgA1 protease. Indeed, in addition to the extensive structural diversity of pneumococcal capsules that undergo shifts over time (2), IgA1 proteases of pneumococci show a significant degree of antigenic diversity (37). To verify this on a broad scale, we compared the genetic diversities of IgA1 proteases among >7,500 pneumococcal isolates, including strains with the same capsular serotype.

**FIG 4:**
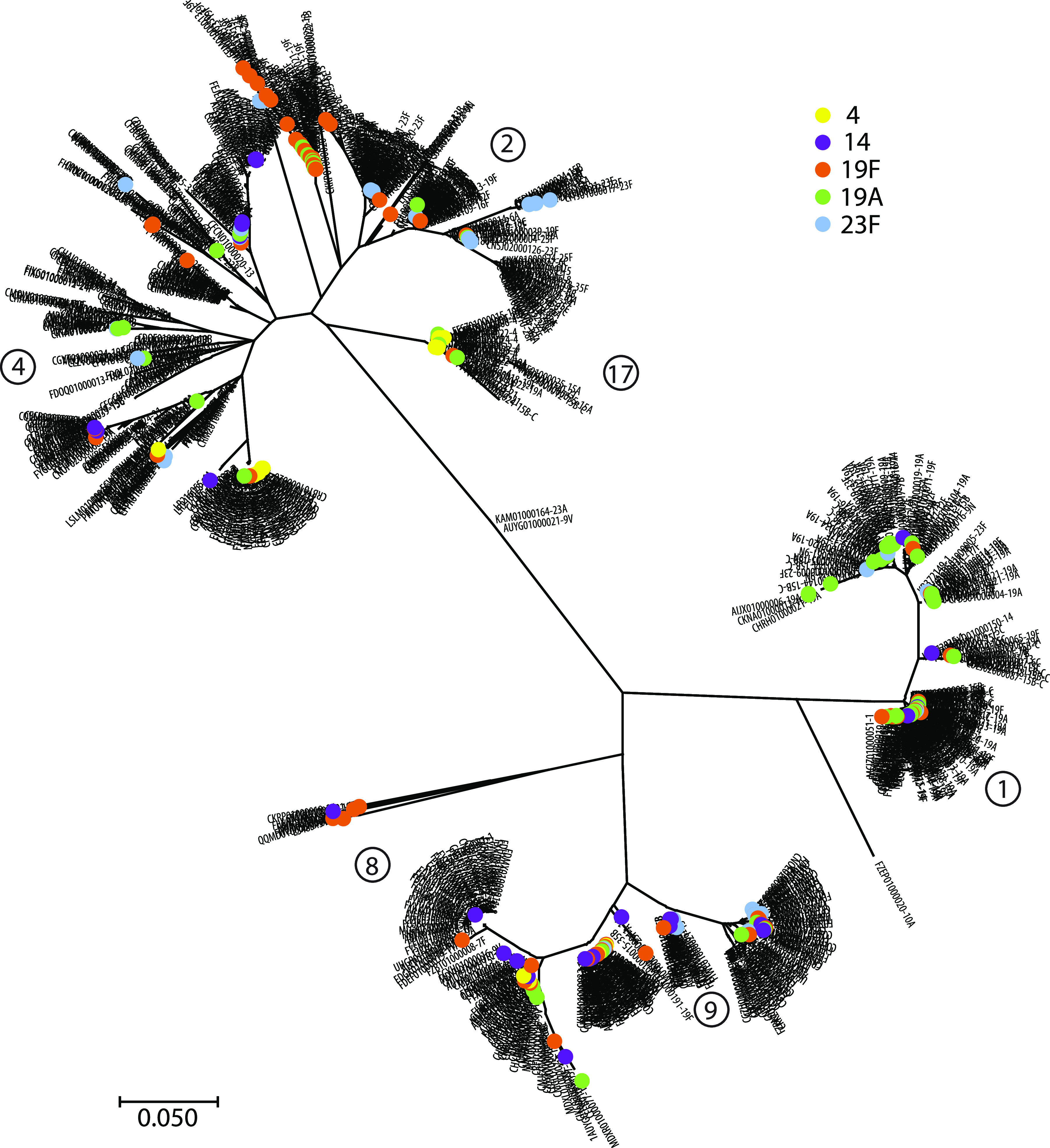
Genetic diversity of *zmpA* genes in 959 representative S. pneumoniae strains of different capsular serotypes. Selected capsular serotypes are indicated by colored dots. Numbers in circles refer to distinct inhibition types of IgA1 proteases detected with neutralizing antibodies raised in rabbits (37).

The phylogenetic tree in Fig. 4 demonstrates a striking genetic diversity of IgA1 protease genes among 959 representative strains of different capsular serotypes. For clarity, five of the serotypes currently included in the 13-valent pneumococcal vaccine Prevnar are highlighted in different colors. The tree includes six *zmpA* genes previously shown to encode IgA1 proteases of distinct inhibitory types (37), which are each harbored on different clades of the tree. This demonstrates that the genetic diversity can be translated into distinct inhibitory types of IgA1 proteases.

## DISCUSSION

From a theoretical point of view, the significance of bacterial IgA1 proteases as important virulence factors is obvious. They enable the producing bacteria to evade the principal humoral immune mechanism, S-IgA1, in the upper respiratory tract. Separation of the monomeric Fab parts from the Fc part, with or without the associated secretory component, negates antibody-mediated agglutination, phagocytosis, and trapping of S-IgA1-coated bacteria in the mucus layer lining the surfaces of the respiratory tract (14–18). The monomeric Fab fragments, released by IgA1 protease activity, retain their antigen-binding capacity (13, 18). Thereby, Fab fragments of IgA1 antibodies enhance bacterial adherence by neutralizing the inhibitory effect of the negatively charged capsules on adhesive interaction with host cells and potentially block access of other isotypes of antibody molecules and their effector functions (16, 43). The specificity of the protease for human IgA1 is in accordance with the host specificity of the three bacterial species. Finally, the convergent evolution of an IgA1 protease in the three principal causes of bacterial meningitis, H. influenzae type b, N. meningitidis, and S. pneumoniae, further corroborates their importance in such infections. Yet, noncapsulated H. influenzae, Neisseria gonorrhoeae, and commensal streptococci related to S. pneumoniae also produce IgA1 proteases, which suggests a more complex association with invasiveness. However, the host specificity of the three pathogens and their respective IgA1 proteases is a substantial hurdle to the development of a relevant *in vivo* model that would provide direct evidence of the exact contribution and mechanism of action of IgA1 proteases in invasive infections. Thus, assuming that the pathogenic events start at the mucosal surfaces, an animal model that actively secretes locally synthesized human IgA1 antibodies would be required. In addition, each of the three pathogens depends on multiple host-specific properties that determine iron acquisition, interactions with host tissues, and virulence. Therefore, in lieu of an animal model, we have to rely on *in vitro* or *ex vivo* assays to support our hypothesis.

Like most other bacterial components and extracellular products, IgA1 proteases induce an immune response. Assays with enzyme-neutralizing antibodies induced in rabbits demonstrated various degrees of intraspecies antigenic diversity (35). Based on experiments that take the antigenic diversity into consideration, our findings, shown in Fig. 1, confirm observations reported by Achtman and coworkers (33, 34) that IgA1 proteases are potent inducers of antibodies in humans colonized by meningococci. Our data clearly demonstrate that such antibodies are capable of neutralizing the enzyme activity in spite of the fact that the enzymes include long stretches of highly diverse repeat regions that may function as immunological decoy mechanisms. Yet, the data in Fig. 1 also show that even high titers of neutralizing antibodies to the IgA1 protease observed in the military recruits that already are or will become colonized do not seem to prevent the acquisition of or sustained colonization by an IgA1 protease-producing pathogen.

These observations raise the crucial question of whether the contribution of the IgA1 protease is restricted to situations in which there are IgA1 antibodies against the surface of the colonizing bacterium—in most cases capsular polysaccharides—and simultaneous absence of neutralizing antibodies to its IgA1 protease (38).

The majority of the human population is intermittently colonized with pneumococci, meningococci, and H. influenzae type b without developing invasive disease (44). Asymptomatic nasopharyngeal carriage is an efficient stimulus for the production of both serum and secretory antibodies to the capsular polysaccharides and other surface antigens, including the IgA1 protease (33, 34, 41, 45–49). As a result, carriage normally confers protection from disease (50, 51). While antibodies to the capsular polysaccharide of meningococci decline rapidly, antibodies to their IgA1 protease do not decline over a 5-year period (34). Several reports noted the apparent paradox that children who develop invasive disease with H. influenzae type b and meningococci have higher titers of both serum and nasopharyngeal antibodies to the organism at the onset of disease than normal controls (39, 52–54). When levels of antibody in acute-phase sera are high, they are predominantly of the IgA class (39). Likewise, several studies noted mucosal S-IgA antibodies to capsular polysaccharide in patients under 23 months of age with H. influenzae type b infections who did not produce and/or sustain a serum antibody level correlated with protection (53, 55).

The most compelling evidence in support of our hypothesis is the observation shown in Fig. 2B. In contrast to controls, the majority (71%) of patients with invasive infection (meningitis and/or septicemia) due to H. influenzae type b and meningococci of serogroups B and C carried microorganisms in the gut that express capsular polysaccharide cross-reactive with the capsule of the respective pathogen. This is in line with two previous series of reports. First, there was the unexpected observation by Ginsburg and coworkers (56) that E. coli K100 was detected in feces of 18 of 92 (19.5%) patients with H. influenzae type b meningitis compared with 2.5% in healthy individuals of comparable age and none of 21 patients with meningitis caused by other bacteria. That study was a follow-up to the demonstrated induction of serum antibodies to the type b capsule in adult volunteers fed cross-reacting E. coli K100 (57), and protective immunity to H. influenzae type b meningitis was achieved by gastrointestinal colonization with E. coli K100 in an infant rat model (58). Second, the reports by Griffiss and coworkers of sporadic cases of N. meningitidis serogroup C disease in patients carrying the cross-reactive E. coli K92 in the gut combined with the demonstration *in vitro* that serum IgA may block the bactericidal activity of IgG (59–61). These observations were sources of inspiration for the generation of our hypothesis.

The basic premise of the model proposed by Griffiss (60) is that “a strain of N. meningitidis expresses its epidemic potential in a population only when it cocirculates with a nonpathogenic enteric organism that elaborates a cross-reacting surface antigen.” The model assumes that IgA antibodies induced by the cross-reacting bacteria lack antibacterial activity and, therefore, have the potential of blocking IgM and IgG antibodies against the pathogen. However, a detrimental role of intact IgA antibodies in the protection against microbial infections is not compatible with comprehensive evidence of the protective potential of IgA. The *in vitro* bactericidal assay employed by Griffiss neglects the protective potential of IgA antibodies mediated by agglutination, trapping of S-IgA-coated bacteria in the mucus layer of mucosal surfaces, and phagocytosis (14–18). Our observations provide further evidence of the cocolonization of the pathogen with cross-reactive bacteria and extend the proposed situation to apply also to H. influenzae type b disease (Fig. 2B). According to our model, the detrimental role of IgA1 antibodies is restricted to pathogens that produce IgA1 protease. In the absence of enzyme-neutralizing antibodies, the protease enables the bacteria to take advantage of the released monomeric Fab fragments of preexisting IgA1 antibodies, which facilitate adherence and efficiently block activities of intact antibodies of other isotypes.

There are several reports on immunologic cross-reactivity of occasional capsular serotypes of S. pneumoniae and capsules of other bacterial species, including the enteric Klebsiella, Escherichia, and *Shigella* spp. and commensal Streptococcus species (62–69). Immunological priming by some of these bacteria may be relevant to invasive S. pneumoniae infection according to our model. This applies primarily to the mentioned enteric bacteria, which are known to induce a strong immune response (57, 70, 71). In contrast, commensal streptococci, and probably other commensal bacteria that live in complete harmony with the host, induce a very limited immune response when acquired and subsequently induce immunological tolerance (72). Our demonstration in Fig. 4 of a multitude of relationships between pneumococcal capsular types and genetically distinct IgA1 proteases suggests that members of the species itself may induce immunological priming for invasive infection too. Nasopharyngeal colonization by S. pneumoniae is a dynamic process, and cocolonization or time-shifted colonization of several genetically distinct clones and members of related commensal streptococci is frequent. This facilitates evolution by recombination and emergence of clones with new combinations of capsular serotype and alleles of other genes, including *zmpA* (3, 21, 73–75). Both the *cps* and *zmpA* loci are recombinational hot spots (2, 3, 74). The sequential mucosal immune response to such clones of pneumococci with identical capsular polysaccharide but distinct IgA1 proteases may set the stage for invasive infection according to our model. Although to a lesser extent, the same mechanism may apply to some cases of meningococcal meningitis, but not to H. influenzae type b because of the conservation of the *zmpA* gene.

Conceivably, the ratio of IgA1 to other isotype antibodies against the capsular polysaccharide and antibodies of any isotype that may neutralize the activity of the IgA1 protease is decisive for the infection process. The level of antibodies to the capsular polysaccharide declines more rapidly (34). It is unknown how long the enhanced susceptibility will last after colonization with the priming cross-reactive bacteria and if a potential secondary and stronger response to the capsule of the pathogen but not to its IgA1 protease is sufficient to maintain the susceptibility. In any case, it is conceivable that colonization with cross-reacting bacteria results in a brief window of susceptibility.

Our study demonstrated the presence of fecal bacteria cross-reacting with the invasive pathogen in more than 70% of examined patients (Fig. 2B). Why are they not detected in all patients with invasive infection due to H. influenzae type b and N. meningitidis serogroups B and C? Apart from potential technical limitations, there are several possible explanations. (i) Our study focused on carriage of cross-reactive microorganisms in the gut. However, we cannot exclude that carriage at other sites (e.g., the upper respiratory tract) may be relevant. In addition to the mentioned genetic variants of S. pneumoniae and potentially N. meningitidis, other bacteria with cross-reacting antigens may be present. For example, the capsule of some clones of Staphylococcus aureus is reported to be identical to that of H. influenzae type b (76). (ii) Relevant bacteria may be undetectable due to antibiotics used in the acute phase of the infection. (iii) According to the hypothesis, the temporal kinetics of the IgA response to the capsule and the inhibitory antibodies to the IgA1 protease is crucial. It is possible that the relevant colonization by the cross-reactive microorganism was terminated at the time of hospitalization. Data from fortnightly nasopharyngeal cultures show that individuals who are admitted with meningococcal disease acquired the pathogen within 1 week of disease onset (77). In addition, several days of preceding illness usually occur before hospitalization.

Conjugate vaccines based on the capsular polysaccharides of H. influenzae type b and selected capsular polysaccharides of meningococci and pneumococci have been successful both in protecting from invasive diseases and in reducing carriage of the respective pathogens (27, 78–80). The conjugated vaccines primarily elicit serum antibodies, and short-lived increases in secretory antibodies occur in approximately 50% of recipients (81–85). The serum antibodies elicited are of all three major immunoglobulin classes. The highest and most sustained increases in adults are of IgG subclass 2. In infants, conjugates elicit booster responses, with the highest increase in IgG1 antibodies followed by IgG2 and, therefore, do not prime for susceptibility to invasive disease according to our model. All vaccine-induced antibodies that reach the mucosae presumably contribute to the observed reduction in carriage of the respective pathogens. According to our model, it is possible that the effect of the vaccines is potentiated by reduced carriage of bacteria expressing capsular polysaccharides cross-reactive with those included in the vaccines. Mucosal vaccination with capsular polysaccharides of the three pathogens conjugated to any surface protein, apart from an IgA1 protease that covers the relevant spectrum of antigenic diversity, would according to our findings, lead to increased susceptibility in humans, though not in an animal model.

N. meningitidis serogroup A is endemic and has been causing cyclic epidemics in sub-Saharan Africa, while it has virtually disappeared from industrialized countries like the United States and those of Europe (5). The serogroup A capsule is composed of repeating units of O-acetylated (α1→6)-linked *N*-acetyl-d-mannosamine-1-phosphate (86). Although structurally distinct, cross-reactions with E. coli K93 and K51, Bacillus pumilus, and S. pneumoniae types 1 and 3 were observed (64, 87, 88). According to our model, the disappearance of N. meningitidis serogroup A disease in industrialized countries may be associated with disappearance of a cross-reactive microorganism due to urbanization.

### Concluding remarks.

**FIG 5:**
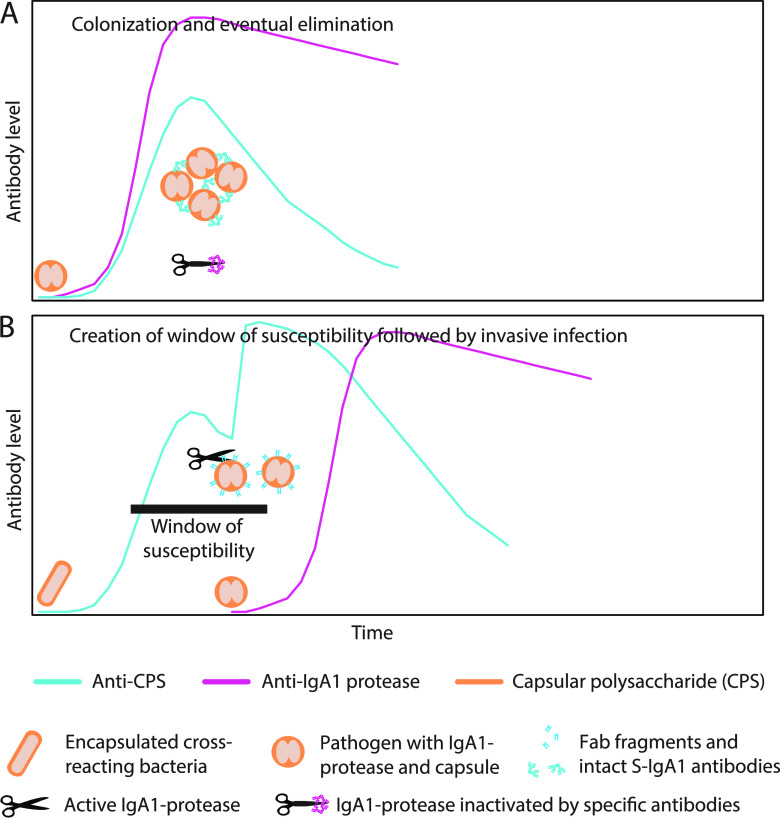
Model for nonsymptomatic colonization (A) and invasive infection (B) with IgA1 protease-producing S. pneumoniae, H. influenzae serotype b, or N. meningitidis. An encounter with the potential pathogen is exemplified by an encapsulated diplococcus. (A) Colonization of upper respiratory tract results in concurrent induction of mucosal IgA antibodies against the capsular polysaccharide (CPS) and the IgA1 protease, immunity, and eventual elimination. Antibodies against the capsular polysaccharide remain intact because antibodies against the IgA1 protease (scissors) inhibit its activity. (B) Colonization with bacteria (exemplified by encapsulated rod) in the gut or respiratory tract (see the text) induces mucosal anti-capsular IgA1 antibodies, which creates a window of susceptibility that may be exploited by a subsequently colonizing pathogen expressing the same capsular polysaccharide. The pathogen may take advantage of the preexisting anti-capsular IgA1 antibodies to coat its surface with Fab fragments released by the IgA1 protease (scissors), which is operative because of the absence of inhibitory antibodies.

The present study provides a spectrum of observations all supporting the hypothesis that the key to invasive disease caused by the three IgA1 protease-producing pathogens S. pneumoniae, N. meningitidis, and H. influenzae type b is induction of anti-capsular IgA1 antibodies prior to the induction of inhibitory antibodies to the IgA1 protease. Priming for disease happens by time-shifted acquisition of two different bacteria, an immunogenic commensal followed by the pathogen, but both expressing the same capsular polysaccharide (Fig. 5). Cleavage of preexisting IgA1 antibodies negates Fc-mediated protective mechanisms and releases capsule-binding monomeric Fab fragments that enhance bacterial adherence and block access of other isotypes of antibody molecules. The model offers an explanation for the rare occurrence of invasive diseases in spite of the comprehensive occurrence of colonization by these three pathogens. However, although substantial, the presented evidence is indirect. Direct evidence would require experimental infection in an animal model, which, in our experience, is not achievable due to extensive host specificity-associated hurdles, including various properties of the pathogen, such as the human-specific IgA1 proteases and other virulence factors.

## MATERIALS AND METHODS

### Examination of bacterial isolates.

Bacterial isolates from pharyngeal swabs of military recruits and from cerebrospinal fluid (CSF) or blood of meningitis or septicemia patients were identified to the species level using standard phenotypic characterization and by capsular serotyping as described previously (89). All isolates were examined for IgA1 protease activity by incubating colonies in a solution of human myeloma IgA1 followed by detection of characteristic cleavage products by immunoelectrophoresis and SDS-PAGE as described previously (90).

### Detection of inhibitory antibodies against IgA1 proteases of colonizing N. meningitidis.

Enrolled in the study were male military recruits who entered Høvelte military camp north of Copenhagen, Denmark, on 1 November 1992. Thirty-six recruits distributed in three groups were included in this part of the study. (i) Persistent noncarriers included 11 recruits who were not detectably colonized with meningococci during the first 3 months of service. (ii) The group with acquisition of N. meningitidis included 11 recruits who became colonized with meningococci within the first 2 weeks of service. (iii) The group of persistent carriers included 14 recruits who were constant carriers of the same strain of meningococcus during the first 3 months of service.

A pharyngeal swab was taken from both tonsils on days 0, 15, 30, 60, and 90 with a charcoal-impregnated, cotton-tipped wooden applicator. To maintain consistency in the sampling procedure, all swabs were taken by one person (J.A.) and inoculated immediately onto a selective chocolate agar medium containing the antibiotics lincomycin (1 μg/mL), amphotericin B (2 μg/mL), polymyxin B sulfate (25 U/mL), and trimethoprim lactate (3 pg/mL) (11). Within 3 to 5 h, all plates were incubated at 36°C in a humid atmosphere plus 5% CO_2_ and observed daily for 3 days. Identification and characterization of presumptive N. meningitidis isolates were performed as described by Andersen et al. (91).

Blood samples were collected from the recruits 1, 2, 7, and 10 weeks after their entry into the camp. To test the hypothesis that colonization induces neutralizing antibodies against the IgA1 protease of the colonizing clone of meningococci, the three groups of samples were analyzed as follows. The IgA1 proteases of meningococcal isolates from carriers were isolated from broth cultures of each of the isolates as described previously (90). Titration of inhibitory serum antibodies against IgA1 protease was performed in an ELISA-based assay measuring released Fab fragments as described previously (92), using activity-calibrated IgA1 protease isolated from the respective meningococcal isolates from carriers and the IgA1 protease of N. meningitidis strain HF13 (noncarriers). We previously identified the latter IgA1 protease as one that induced inhibitory antibodies against the entire spectrum of meningococcal and gonococcal IgA1 proteases when injected into rabbits (36).

The same procedure was used to measure inhibitory antibodies to the respective IgA1 proteases of meningococcal isolates from infected patients.

### Gut carriage of bacteria expressing polysaccharides cross-reacting with pathogen.

During a period of 3 years (November 1993 to September 1996), patients with meningitis/septicemia caused by H. influenzae type b or N. meningitidis admitted to selected university-associated Danish hospitals were enrolled in the study. Age-matched patients examined at the respective hospitals for other reasons served as controls. All cerebrospinal fluid (CSF) or blood isolates were received from the clinical bacteriology laboratory as the first subculture after primary isolation. These were streaked on chocolate agar (Statens Serum Institut, Copenhagen, Denmark) and incubated overnight at 37°C in air plus 5% CO_2_. Serological capsule typing was confirmed by slide agglutination using capsular typing antisera, and the remainder of the second subculture was suspended in sterile skim milk and frozen at −70°C.

From each subject, blood, saliva, and fecal samples were collected as soon after admission as possible. In connection with the diagnostic blood sample, an extra 5 mL of blood was collected into a glass without anticoagulant. Fecal samples were collected with a feces spoon. If possible, samples of saliva were collected using the Omni-SAL collection device (Saliva Diagnostic Systems, Singapore, Pte, Ltd.) according to the instructions provided by the company. Transported from the regional hospital, the samples reached the laboratory at Aarhus University on the day of collection or within 1 day. The feces sample was transferred to a tube with 1 mL of 0.01 M phosphate-buffered saline (PBS) buffer with 0.154 M NaCl, suspended, weighed, and used for two purposes. For the detection of the presence of microbial antigens cross-reactive with the capsular polysaccharide of the respective pathogen, the sample was heat inactivated at 60°C for 20 min and then examined in double-immunodiffusion assays (Fig. 2A). Suspensions of heat-inactivated H. influenzae type b, N. meningitidis serogroup B, N. meningitidis serogroup C, and E. coli capsular serotypes K1, K92, and K100 served as controls. The Hib burro antiserum was a gift from Rachel Schneerson, NIH, Bethesda, MD, USA, and the anti-N. meningitidis group B and anti-N. meningitidis group C sera were purchased at Statens Serum Institut.

The feces suspensions were streaked onto brain heart infusion agar, MacConkey agar, and chocolate agar with bacitracin (300 mg/L) for selective culture of Haemophilus spp., as well as an in-house medium for cultivation of anaerobic bacteria consisting of Columbia agar base (42.5 g) (BD), hemin (5 mg), and vitamin K solution (10 mg/L of water) (Konakion Novum). After cooling to 50°C in a water bath, 50 mL defibrinated horse blood was added. After incubation for 48 h in air plus 5% CO_2_ or in an anaerobic chamber (supplemented Columbia agar), 10 colonies of distinct morphology were isolated from each agar medium, purified by subcultivation on the same medium, and checked for cross-reactive surface antigens by double immunodiffusion as described above.

### Quantitation of serum and secretory antibodies to capsular polysaccharide.

Antibodies to the group B capsular polysaccharide of N. meningitidis were measured by ELISA. Purified polysaccharide of N. meningitidis group B was purchased from Pasteur Mérieux Connaught (Marcy l’Étoile, France). The phenylated polysaccharide used for coating of the ELISA plates was prepared as described by Konradsen et al. (93). Briefly, the polysaccharide was activated with cyanogen bromide at pH 10.5 for 2 min. The spacer 2-phenylethylamine was added, and the pH was adjusted to 9.0. After coupling for 2 h, the pH was adjusted to 5.0 with acetic acid and the polysaccharide derivatives precipitated with ethanol.

Briefly, the ELISA was performed as follows. Ninety-six-well MaxiSorp plates (Nunc, Roskilde, Denmark) were coated overnight with 100 μL/well (0.1 μg) of phenylated group B polysaccharide. After blocking for 2 h at room temperature with PBS–0.05% Tween 20 supplemented with 10% fetal calf serum (FCS), duplicates of serial dilutions of sera or salivas as well as positive controls in PBS–Tween–1% FCS were added to the plates. Following overnight incubation at 4°C, 100 μL/well of biotin-labeled monoclonal mouse IgG1, anti-human IgA1 (Sigma), or rabbit anti-human immunoglobulin (Dakopatts) at 1:2,000 dilution was added, and plates were incubated for 2 h at room temperature. After being washed with PBS-Tween, a 1:3,000 dilution of alkaline phosphatase-labeled streptavidin (Sigma) was added to each well. Following incubation for 2 h at room temperature, 100 μL/well of enzyme substrate (1 mg/mL 4-dinitrophenylsulfate-Na salt in 1 M diethanolamine 1 mM MgCl_2_) was added. The color was allowed to develop for 1 h at room temperature and stopped with 1 M sodium hydroxide, and the optical densities were measured at 405 nm (OD_405_) with an ELISA reader. The results were expressed as endpoint titers, which are the reciprocal of the highest dilution of sera or saliva that had an OD higher than the cutoff value (OD over 2 background values).

### Genetic shift of IgA1 protease allele in S. pneumoniae serotypes.

A set of six canonical *zmpA* nucleotide sequences (ATCC700669_SPN23F10580, GSP14_SPCG_RS05920, G54_SPG_RS05310, P1031_SPP_RS05660, D39_SPD_1018, and TIGR4_SP_1154) were used for a blastn search of the database of >7,500 streptococcal genomes described by Kilian and Tettelin (74). Alignments of genome sequences with ≥60% identity over ≥40% of the query sequence length were selected, and a single copy of loci with hits from multiple queries was kept. This resulted in the identification of 7,917 loci with similarity to *zmpA*. Because this number of sequences was too large for multiple-sequence alignment, a pairwise sequence distance matrix was calculated using MASH v2.1 (https://www.ncbi.nlm.nih.gov/pubmed/27323842). The matrix included reference sequences of pneumococcal *zmpB*, *zmpC*, and *zmpD* to verify the identity of the *zmpA* genes. The matrix was then converted to .meg format using an in-house script for processing with megacc 10.0.5 (https://pubmed.ncbi.nlm.nih.gov/22923298/) to compute a neighbor-joining tree using pairwise deletion. A Newick output was then loaded into Dendroscope 3.7.4 (https://pubmed.ncbi.nlm.nih.gov/22780991/) to generate a midpoint-rooted circular phylogram. Strain serotype information was extracted from the PubMLST database (https://pubmlst.org/bigsdb?db=pubmlst_spneumoniae_isolates) (15 July 2020) when available. When unavailable, *in silico* serotyping of whole-genome sequences was performed on Illumina fastq files using seroBA (https://pubmed.ncbi.nlm.nih.gov/29870330/); seroBA commands getPneumocat and createDBs with a k-mer size set at 71 were run on 3 November 2020 to create the reference databases. For assembled genomes where no fastq file was available, genome assemblies were converted to fastq files using the BBMap/BBTools 38.47 (Ref.) shred.sh utility (parameters: length = 150, minlength = 150, overlap = 149); the output fastq files were then subjected to seroBA.

Two representatives of strains of distinct capsular serotype in each cluster in the phylogram were selected for detailed cluster analysis. The resultant subset of 959 pneumococcal *zmpA* sequences were aligned using muscle 3.7 (94), and the multi-fasta file of aligned sequences was loaded into MegaX (95) for manual editing and for generation of an .nwk file with branch lengths, which was loaded into Dendroscope 3.7.4 for generation of a circular tree.

### Ethical procedure.

The carrier study in military recruits was performed after permission from the Local Committee of Ethics in Science. The permit for the study of meningitis patients was obtained from the Committee of Ethics in Science for the Aarhus region (reference 1993/2600), with permission extended to all Danish regions. Participation was on a voluntary basis and in accordance with the guidelines of the Helsinki II declaration. In cases of meningitis patients, informed consent was obtained from parents or the closest relative.

### Statistical procedures.

Normality was determined using the Shapiro-Wilks W test and visually inspected using Q–Q-plots. In case of nonnormality, continuous and categorical variables were analyzed using the Mann-Whitney U test and the X2 test, respectively. In case of normality, Student’s *t* test was used. The alpha level for all tests was 0.05. Statistical analyses were performed using STATA/IC 16·1 (StataCorp LP, Texas).
